# Aberrant regeneration of third nerve with characteristic lid signs: operating the normal fellow eye

**DOI:** 10.22336/rjo.2021.80

**Published:** 2021

**Authors:** Sumita Sethi

**Affiliations:** * Incharge Pediatric Ophthalmology and Strabismus Services. BPS GMC for Women, Khanpur, Sonepat, Haryana, India

**Keywords:** third nerve palsy, aberrant regeneration, inverse-Duane’s sign, pseudo-Graefe’s sign

## Abstract

A young female with old traumatic third nerve palsy presented with characteristic lid signs of aberrant regeneration of third nerve. There was noticeable disparity in the lid aperture and surgery on the normal fellow eye was undertaken to offer the patient a satisfactory aesthetic appearance.

## Introduction

Aberrant regeneration of third nerve is known to occur most frequently following traumatic head injury and aneurysm of posterior carotid artery [**1**]. Full blown features of the syndrome might not be present in each case but the lid signs are characteristic and important clues for diagnosis [**2**]. The most common lid signs include horizontal gaze-eyelid synkinesis, pseudo Graefe (Fuch’s) sign, adduction of eye on attempted vertical movement with retraction of globe on attempted vertical movement and Pseudo Argyll-Robertson pupil. In our case, noticeable changes were observed in eye aperture; large amount of EOM surgery may possibly have worsened the lid aperture disparity; surgery on the fellow eye was undertaken for better aesthetic appearance.

## Case report

A 16-year-old female presented with complaint of out deviation of the right eye, lid aperture changes and severe dimness of vision OD for the last 10 years. History revealed head trauma 10 years back. Afterwards, the patient developed complete dropping of upper lid, outward deviation, and acute onset of severe dimness of vision OD. The dropping of upper lid gradually improved over a period of 3 months. There were no complaints for OS and no systemic complaints at all. 

**Fig. 1:**
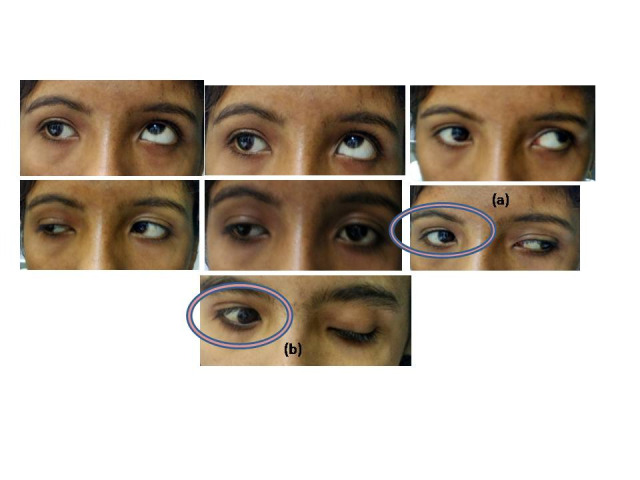
Pre-operative photographs showing exotropia in primary gaze with lid signs; (a) horizontal gaze-eyelid synkinesis (b) pseudo-Graefe sign

The imaging of brain revealed no significant abnormality. Complete ophthalmological examination was undertaken including refraction, torch light examination, extraocular movements and Krimsky test. BCVA OD was finger counting 3-4 feet; afferent pupillary defect was present. In primary gaze, right exotropia (50 D) and hypotropia (8D) were present by Krimsky test with mild ptosis. Supraduction was absent, infraduction was limited OD and characteristic elevation of right upper lid on adduction (gaze-eyelid synkinesis) and retraction and elevation of upper eyelid on downgaze (pseudo-Graefe sign) were present (**Fig. 1**). Fundoscopy was normal OD; ophthalmic examination OS was unremarkable. There was no definite explanation for the severely diminished vision OD since old records, especially imaging at the time of trauma, were missing; the most probable explanation being direct or indirect damage to optic nerve at the time of trauma.

**Fig. 2:**
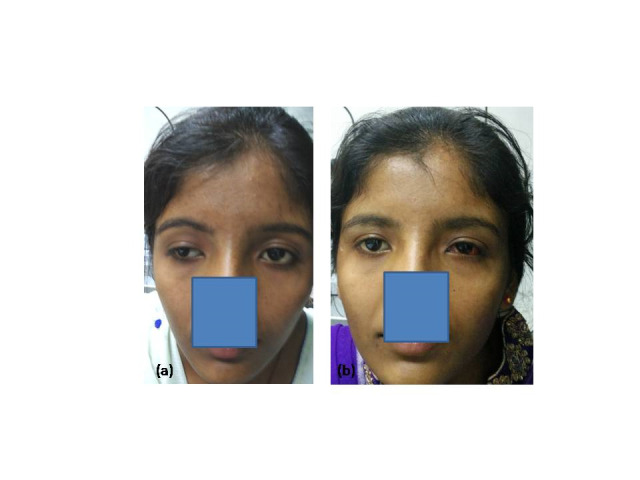
(a) Pre-operative and (b) immediate post-operative photograph showing acceptable aesthetic appearance with concern to lid disparity

**Fig. 3:**
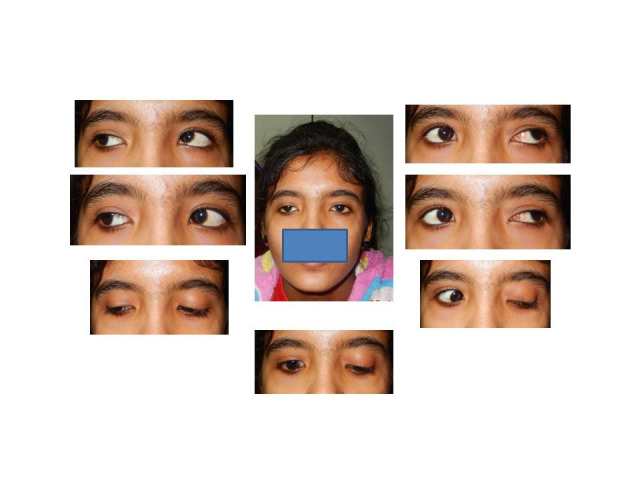
Six months postoperative photograph showing persistence of lid synkinesis in lateral gaze but improved appearance in primary gaze

A diagnosis of long standing traumatic third nerve palsy with aberrant regeneration was established. In lieu of the significant lid aperture changes it was realized that surgery on the same eye might worsen the aperture disparity. Therefore, recession and resection of the fellow eye was planned. Lateral rectus recession of 10.0 mm and medial rectus resection of 7.0 mm were undertaken keeping a target angle of 50 D. A reasonable aesthetic appearance was achieved on the first operative day (**Fig. 2**). Mild residual XT of 6 D with satisfactory appearance was observed at 6 months follow-up (**Fig. 3**).

## Discussion

The phenomenon of aberrant regeneration of third nerve being caused by misdirected regenerating third nerve fibers is uncommon but has been well described in literature and documented by ocular electromyography. Chua HC et al. reported three patients with aberrant regeneration of third nerve following traumatic brain injury and concluded that full blown features may or may not be present; the misdirection incidence in their series was 15% [**3**]. 

In a retrospective series of 46 operated patients for third nerve palsy, the authors observed that the success rate was unaffected by the presence of aberrant regeneration [**4**]. They also found a slightly higher success rate with adjustable versus nonadjustable sutures. However, while pre-operatively assessing this case, it was realized that the difference in lid aperture in primary gaze was an important concern. Moreover, synkinesis in adduction could not have been well treated, but the young female was concerned for her cosmetic appearance in the primary gaze. Performing any large EOM surgery in the palsied eye might have worsened the lid aperture disparity in the primary gaze. A surgery in the fellow eye was thus undertaken in the form of large recession and resection resulting in satisfactory aesthetic appearance in the primary gaze. Also, the adjustable technique was not used since visual acuity was already much compromised and a more than full correction was required.

Surgical management of acquired third nerve palsy depends on the number and extent of involvement of extra ocular muscles as well as the presence or absence of signs of aberrant regeneration [**5**]. Various methods have been described; large recess resection procedures have been time tested; newer globe fixation techniques in the form of periosteal fixation and medial transposition of split lateral rectus muscle [**6**] have also been successfully done in third nerve palsy patients. While functional results were not very good, in most cases, the aim was to provide acceptable aesthetic appearance. In 2003, Parulekar MV introduced for the first time the concept of operating the fellow eye to restore ocular alignment [**7**]. Our results further strengthen the idea of making use of the non-paretic eye for managing the lid aperture disparity. However, at present, a further modification in the form of adjustable suture would be a better procedure. But this was not required in our case.

## Conclusion

In cases of acquired third nerve palsy, it becomes important to recognize clinical signs of aberrant regeneration and decide the surgical procedure accordingly. Disparity in lid aperture could be a concern in some patients, whose condition might worsen by performing EOM surgery on that eye. Yet, the surgery of the fellow eye was a viable option.

**Conflict of Interest statement**

The authors state no conflict of interest.

**Informed Consent and Human and Animal Rights statement**

Informed consent has been obtained from all individuals included in this study.

**Authorization for the use of human subjects**

Ethical approval: The research related to human use complies with all the relevant national regulations, institutional policies, is in accordance with the tenets of the Helsinki Declaration, and has been approved by the review board of Pediatric Ophthalmology and Strabismus Services, BPS GMC for Women, Khanpur, Sonepat, Haryana, India.

**Acknowledgements**

None.

**Sources of Funding**

None.

**Disclosures**

None.
